# Associations of dietary macronutrients and micronutrients with the traditional and nontraditional risk factors for cardiovascular disease among hemodialysis patients: A clinical cross-sectional study

**DOI:** 10.1097/MD.0000000000011306

**Published:** 2018-06-29

**Authors:** Tuyen Van Duong, Te-Chih Wong, Chien-Tien Su, Hsi-Hsien Chen, Tzen-Wen Chen, Tso-Hsiao Chen, Yung-Ho Hsu, Sheng-Jeng Peng, Ko-Lin Kuo, Hsiang-Chung Liu, En-Tsu Lin, Shwu-Huey Yang

**Affiliations:** a School of Nutrition and Health Sciences, Taipei Medical University; b Department of Nutrition and Health Sciences, Chinese Culture University; c School of Public Health, Taipei Medical University; d Department of Family Medicine; e Department of Nephrology, Taipei Medical University Hospital; f School of Medicine, Taipei Medical University; g Department of Nephrology, Taipei Medical University-Wan Fang Hospital; h Division of Nephrology, Department of Internal Medicine, Taipei Medical University Shuang Ho Hospital, Taipei Medical University; i Division of Nephrology, Cathay General Hospital; j Division of Nephrology, Taipei Tzu-Chi Hospital, Taipei; k Department of Nephrology, Wei Gong Memorial Hospital, Miaoli; l Department of Nephrology, Lotung Poh-Ai Hospital, Yilan; m Nutrition Research Center, Taipei Medical University Hospital; n Research Center of Geriatric Nutrition, Taipei Medical University, Taipei, Taiwan.

**Keywords:** cardiovascular disease, dietary intake, hemodialysis patients, macronutrients and micronutrients, traditional and nontraditional risks

## Abstract

The current study was to examine the association of adequate intake of macronutrients and micronutrients with traditional and nontraditional cardiovascular risk factors in hemodialysis patients.

A clinical cross-sectional study was conducted between September 2013 and April 2017 on 492 hemodialysis patients aged 20 years and above, received thrice-weekly hemodialysis treatment for at least 3 months, adequate dialysis quality (equilibrated Kt/V ≥ 1.2 g/kg/d) from 7 hospital-based hemodialysis centers in Taiwan. The dietary intake was evaluated by the 3-day dietary record, and a 24-hour dietary recall. Biochemical parameters were archived from laboratory tests. The cardiovascular disease (CVD) risk factors were defined by the Kidney Disease Outcomes Quality Initiative (K/DOQI) Clinical Practice Guidelines. The adequate dietary intake of macronutrients and micronutrients was recommended by the European Best Practice Guidelines, K/DOQI, and Institute of Medicine guidelines. Logistic regression analysis was used.

All hemodialysis patients had CVD risks, the lowest proportion of patients with adequate intake of macronutrients and micronutrients were 8.7% and 1.8%, respectively. The adequate dietary intake associated with lower likelihood of having CVD risks in hemodialysis patient by 47% to 84%, including 39% to 58% lower hypertension, 37% to 50% lower dyslipidemia, 42% to 63% diabetes mellitus, 44% to 84% lower obesity, 58% lower low calcium, 38% lower hyperparathyroidism, 47% to 64% lower hyperhomocysteinemia, and 41% to 67% lower inflammation, 63% to 74% lower hypoalbumin, 73% lower inadequate normalized protein nitrogen appearance.

Adequate dietary nutrients intake may reduce the cardiovascular risks factors, in turn, to prevent the cardiovascular morbidity and mortality.

## 1 Introduction

Cardiovascular diseases (CVDs) are the leading cause of death in patients with end-stage renal disease, and contribute to more than half of all deaths.^[1]^ In Taiwan, the CVD account for 100% higher mortality in chronic kidney disease, as shown in a prospective cohort study.^[2]^

Patients with chronic kidney disease are in the high-risk group for cardiovascular events and diseases.^[3]^ The traditional CVD risks (older age, men, hypertension, dyslipidemia, diabetes, obesity, and inactivity), and nontraditional CVD risks (hyperhomocysteinemia, chronic inflammation, anemia, mineral metabolic abnormalities, malnutrition, electrolyte imbalance) are summarized by Sarnak and colleagues.^[3,4]^ The CVD risk factors are also defined by Kidney Disease Outcomes Quality Initiative (K/DOQI) Clinical Practice Guidelines for Cardiovascular Disease in Dialysis Patients.^[5]^ These traditional and nontraditional/novel risk factors strongly associated with cardiovascular events and mortality among patients with chronic kidney disease.^[6,7]^

The multifactorial intervention strategies targeted on traditional and nontraditional CVD risk factors are required for early prevention of CVD, including pharmacologic, nutritional, and lifestyle approaches.^[8]^ Nutritional interventions show the potential impacts on the better clinical outcomes, lower mortality rate, other health benefits, lower hospitalization rate, and expenditure among hemodialysis patients.^[9,10]^ However, the majority of hemodialysis patients do not meet the dietary requirements to reduce the CVD risk factors,^[11]^ while nonadherence to dietary regimen can lead to adverse clinical outcomes, increase morbidity and mortality.^[12]^

The role of dietary intake on CVD risk factors is remained to be investigated in hemodialysis patients. This study examines the association of macronutrient and micronutrient intakes with traditional and nontraditional CVD risk factors among hemodialysis patients in multiple dialysis centers in Taiwan. We hypothesize that patients consumed adequate nutrients had a lower likelihood of having CVD risk factors.

## 2 Methods

### 2.1 Study design

We conducted a clinical cross-sectional study between September 2013 and April 2017 on 492 hemodialysis patients in 7 hemodialysis centers in Taiwan, including those in Taipei Medical University Hospital, Taipei Medical University – Wan Fang Hospital, Taipei Medical University – Shuang Ho Hospital, Cathay General Hospital, and Taipei Tzu-Chi Hospital, Wei-Gong Memorial Hospital, and Lotung Poh-Ai Hospital.

### 2.2 Study population

The study was conducted on patients who aged above 20 years, received thrice-weekly hemodialysis treatment for at least 3 months, adequate dialysis quality (equilibrated Kt/V ≥ 1.2 g/kg/d). Patients who diagnosed with edema, pregnancy, amputation, hyperthyroidism, hypothyroidism, malignancy, received tube feeding, exhibited hepatic failure or cancer, hospitalized within 1 month prior to the recruitment, or scheduled for surgery were excluded.

The eligible patients participated in the interviews (by face-to-face and telephone) conducted by qualified dietitians in selected hospitals. The informed consent form was signed by patients before conducting interview and examinations. The patients’ medical records were reviewed. The blood samples were collected by licensed nurses, at the start of the first dialysis session of the week, then analyzed in the hospital laboratory by using commercially available test kits, which was described carefully in previous studies.^[13,14]^

### 2.3 Assessment of CVD risk factors

#### 2.3.1 Traditional CVD risk factors

The risks of cardiovascular events and diseases are older age, men gender, and following factors.^[4,15]^ Hypertension: systolic blood pressure ≥ 130 mm Hg, and diastolic blood pressure ≥ 85 mm Hg^[5]^; diabetes mellitus: patients diagnosed with type 2 diabetes mellitus or fasting plasma glucose ≥ 100 mg/dL^[5]^; dyslipidemia which is suggested by Expert Panel on Detection Evaluation and Treatment of High Blood Cholesterol in Adults including, high serum triglyceride (TG) level at TG ≥ 150 mg/dL; low level of serum high-density lipoprotein cholesterol (HDL-C) at <40 mg/dL in men, and <50 mg/dL in women; high level of serum low-density lipoprotein cholesterol (LDL-C) at ≥100 mg/dL, high serum total cholesterol (TC) at ≥200 mg/dL^[16]^; obesity was defined as body mass index (BMI) ≥ 27.0 kg/m^2^ as recommended by Ministry of Health and Welfare in Taiwan.^[17]^

#### 2.3.2 Nontraditional/novel CVD risk factors

Anemia: The targeted hemoglobin (Hb) level should be 11 g/dL or greater, as moderately strong recommended by The National Kidney Foundation K/DOQI Work Group.^[18]^ Anemia is classified as Hb < 11 g/dL. Mineral metabolism abnormalities: Albumin-corrected calcium = total calcium (mg/dL) + 0.8 × (4.0 – serum albumin in g/dL).^[19]^ Corrected calcium and phosphorus levels at each time were used to calculate calcium–phosphorus product (Ca × PO_4_). The serum calcium is classified into low level (Ca < 8.4 mg/dL), normal level (Ca = 8.4–9.5 mg/dL), and high (Ca > 9.5 mg/dL). The serum phosphorus (PO_4_) is also classified into low level (PO_4_ < 3.5 mg/dL), normal (PO_4_ = 3.5–5.5 mg/dL), and high (PO_4_ > 5.5 mg/dL). Calcium–phosphorus product is classified into normal (Ca × PO_4_ < 55 mg^2^/dL^2^), and high (Ca × PO_4_ ≥ 55 mg^2^/dL^2^). In addition, intact parathyroid hormone (iPTH) is classified as normal (iPTH = 150–300 pg/mL), and high (iPTH ≥ 300 pg/mL).^[20]^ Hyperhomocysteinemia is defined as total plasma homocysteine > 14 μmol/L.^[15]^ Inflammation is defined as high-sensitive C-reactive protein (hs-CRP) > 0.3 mg/dL as the risk factor for CVD.^[21]^ The poor nutritional status is defined as serum albumin ≤ 3.5 mg/dL, serum creatinine ≤ 7.5 mg/dL, and normalized protein nitrogen appearance (nPNA) < 1.0 g/kg as applied in hemodialysis patients from 11 countries in the Dialysis Outcomes and Practice Patterns Study.^[22]^ Hyperkalemia is identified as serum potassium ≥ 5.0 mEq/L as the risk of cardiovascular mortality in hemodialysis patients.^[23]^

### 2.4 Dietary intake assessments

The dietary intake of patients was evaluated by a 3-day dietary record (1 day of hemodialysis, 1 day of nonhemodialysis, and 1 day in the weekend). The dietitians then used the 24-hour dietary recall with common household measuring utensils as the means to confirm the data, which described in details elsewhere.^[13,14]^ Nutrients were then analyzed using the e-Kitchen software (Nutritionist Edition, Enhancement plus 3, version 2009, Taichung, Taiwan).

The application of specific guidelines for renal disease is used, including the European Best Practice Guideline on Nutrition and Chronic Kidney Disease,^[24]^ the guidelines of National Kidney Foundation-K/DOQI for Nutrition in Chronic Renal Failure,^[25]^ and the Standing Committee on the Scientific evaluation of Reference Intakes from Scientific Evaluation of Dietary Reference Intakes, Food and Nutrition Board, Institute of Medicine in United States of America.^[26]^

Macronutrients: The guidelines of K/DOQI recommend that the optimal targets for dietary protein and energy in maintenance hemodialysis patient are ≥1.2 g/kg of ideal body weight/d, ≥35 kcal/kg/d if age <60 years, and ≥30 kcal/kg/d if age ≥60 years, respectively.^[25,27]^ The ideal body weight in the present study is calculated from the height and a BMI of 22, as its validity in hemodialysis patients.^[28]^ It is recommended to consume total fat should not exceed 30% energy intake, reducing dietary intake saturated fat (<10% total energy), monounsaturated fat (≤20% total energy), polyunsaturated fat (≤10% total energy), and cholesterol intake (<200 mg/d).^[29]^ Daily carbohydrate intake should reach 45% to 65% total energy, and fiber intake ≥20 g/d.^[25,27,30]^

Micronutrients: The daily recommended dietary intake of minerals is 1800 to 2500 mg sodium, 2000 to 2500 mg potassium, 800 to 1000 mg phosphorus, 500 to 800 mg calcium, 200 to 300 mg magnesium, ≥8 mg iron (≥18 mg/d for women ≤50 years old), ≥10 mg zinc for men, ≥8 mg zinc for women, and usually 750 to 1500 mL water. The daily dietary vitamins intake is targeted with vitamin B_1_ ≥ 1.2 mg for men, ≥1.1 mg for women; vitamin B_2_ ≥ 1.3 mg for men, ≥1.1 mg for women; niacin (vitamin B_3_) ≥ 16 mg for men, ≥14 mg for women; vitamin B_6_ ≥ 1.3 mg for age ≤ 50 years, ≥1.7 mg for men and ≥1.5 mg for women with age >50 years; folic acid (vitamin B_9_) ≥ 400 μg; vitamin B_12_ ≥ 2.4 μg; vitamin C ≥ 90 mg for men, ≥75 mg for women; vitamin A ≥ 900 μg for men, ≥700 μg for women; vitamin D ≥ 5 μg, ≥10 μg, ≥15 μg for age ≤ 50 years, 50 to 70 years, and >70 years, respectively; and finally, vitamin E ≥ 15 mg.^[24,26]^

### 2.5 Ethical approval

The study was approved by Taipei Medical University Joint Institutional Review Board (TMU-JIRB no 201302024), which was for 3 hospitals of Taipei Medical University (Taipei Medical University Hospital, Wan-Fang Hospital, Shuang Ho Hospital), Wei-Gong Memorial Hospital, and Lotung Poh-Ai Hospital, Cathay General Hospital (CGH-OP104001), and Taipei Tzu-Chi Hospital (04-M11-090). All patients involved in the study have signed the informed consent statement.

### 2.6 Statistical analysis

The descriptive analyses describe the status of nutrients intake, and CVD risk factors via the mean, standard deviation, or mean (minimum, maximum), median, interquartile range, frequency and percentage of studied variables. To carefully examine the association of adequate dietary macronutrients and micronutrients intake with traditional and nontraditional risk factors, the multivariate logistic regressions are used (adjusted for age and gender, hemodialysis vintage, Charlson comorbidity index) to estimate the odds ratios (ORs). All statistical analyses are performed by the SPSS for Windows version 20.0 (IBM Corp, New York, NY). The significant level is set at *P*-value < .05.

## 3 Results

Table 1 shows the proportion of different CVD risk factors which are 38.2% aged ≥65 years, 56.3% men, 81.9% hypertension, 18.7% high TC, 48.4% high LDL-C, 65.9% low HDL-C, 42.7% high TG, 68.7% diabetes, 15.2% obesity. The nontraditional CVD risk factors are with 58.3% anemia, 8.5% low serum calcium, 36.0% high serum calcium, 8.1% low serum phosphorus, 33.5% high serum phosphorus, 25.0% high calcium phosphorus product, 42.5% hyperparathyroidism, 85.7% hyperhomocysteinemia, 50.5% high hs-CRP, 11.4% hypoalbuminemia, 6.3% low serum creatinine, 11.0% low nPNA, and 32.5% hyperkalemia. All hemodialysis patients have risk factors for cardiovascular events or diseases range from 3 to 15 risks.

**Table 1:**
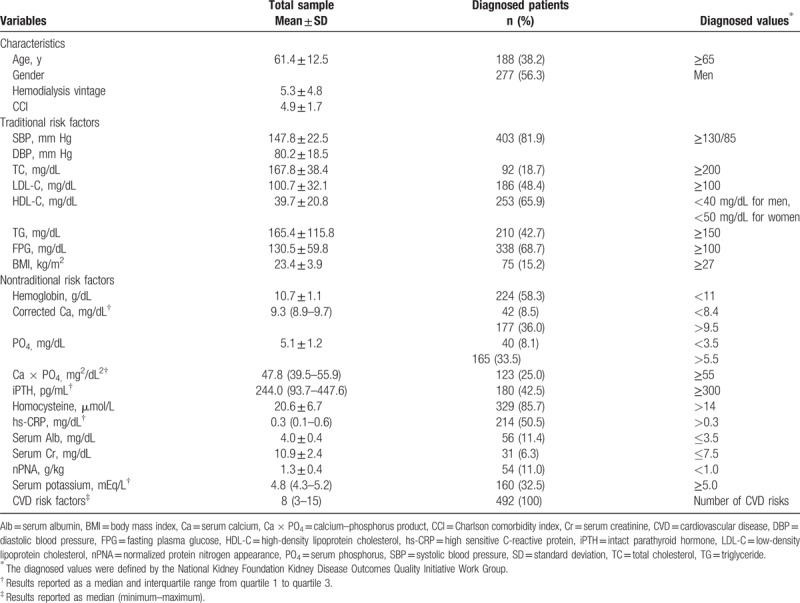
Characteristics, traditional, and nontraditional cardiovascular risk factors of hemodialysis patients (n = 492).

Table 2 demonstrates the prevalence of adequate macronutrients intake which are 23.2% total energy, 27.8% protein, 54.9% carbohydrate, 15.2% total fat, 49.0% cholesterol, and 8.7% total fiber. The adequate minerals and water consume are 6.9% sodium, 6.7% potassium, 12.2% phosphorus, 6.3% calcium, 10.0% magnesium, 34.1% iron, 22.6% zinc, and 29.9% water. Finally, the adequate vitamin intakes are 13.4% vitamin B_1_, 13.2% vitamin B_2_, 18.1% niacin, 11.2% vitamin B_6_, 1.8% folic acid, 53.0% vitamin B_12_, 41.9% vitamin C, 40.4% vitamin A, 3.1% vitamin D, and 20.9% vitamin E.

**Table 2:**
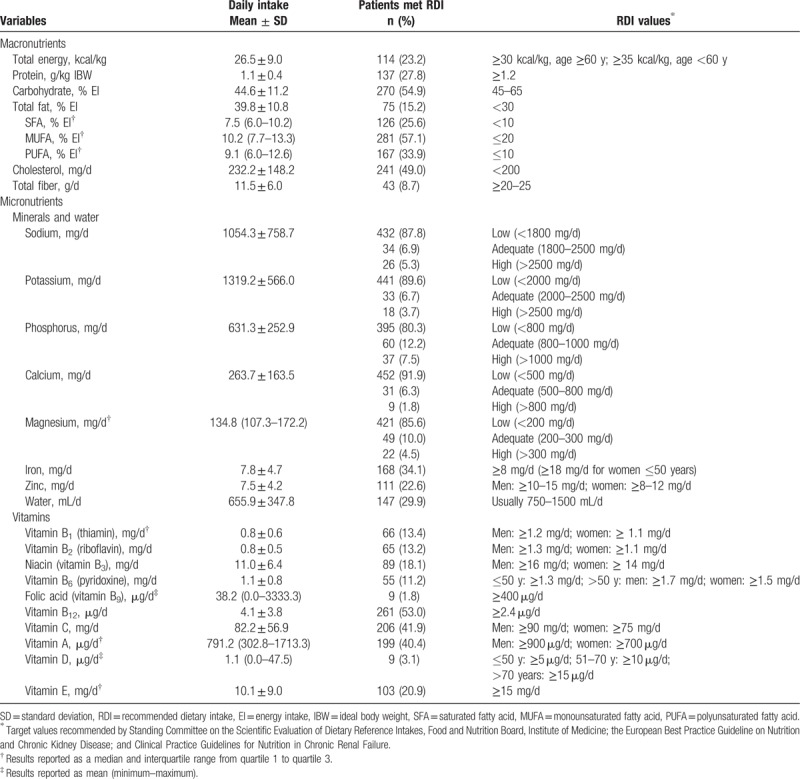
Daily macronutrients, micronutrient intake of hemodialysis patients and prevalence of individual within recommended targets (n = 492).

Table 3 illustrates results of multivariate logistic regression analysis which show the adequate intake of dietary nutrients significantly relate to lower odds of hypertension are saturated fatty acid (SFA) intake (OR, 0.47; 95% confidence interval [95% CI], 0.28–0.78, *P* = .004), monounsaturated fatty acid (MUFA) intake (OR, 0.58; 95% CI, 0.35–0.96; *P* = .033), polyunsaturated fatty acid (PUFA) intake (OR, 0.61; 95% CI, 0.38–0.98; *P* = .043), total fiber intake (OR, 0.42; 95% CI, 0.21–0.85; *P* = .016); lower odds of high LDL-C are cholesterol intake (OR, 0.63; 95% CI, 0.41–0.97; *P* = .034), zinc intake (OR, 0.59; 95% CI, 0.36–0.98; *P* = .040), and vitamin E intake (OR, 0.56; 95% CI, 0.35–0.90; *P* = .016); lower odds of high TG are total energy intake (OR, 0.58; 95% CI, 0.37–0.92; *P* = .019), cholesterol intake (OR, 0.62; 95% CI, 0.43–0.91; *P* = .014); lower odds of diabetes mellitus are total energy intake (OR, 0.37; 95% CI, 0.23–0.59; *P* < .001), SFA intake (OR, 0.47; 95% CI, 0.30–0.76; *P* = .002), MUFA intake (OR, 0.37; 95% CI, 0.24–0.58; *P* < .001), vitamin A intake (OR, 0.58; 95% CI, 0.38–0.87; *P* = .009); to lower odds of obesity are total energy intake (OR, 0.16; 95% CI, 0.05–0.44; *P* < .001), PUFA (OR, 0.53; 95% CI, 0.30–0.95; *P* = .034), cholesterol intake (OR, 0.56; 95% CI, 0.33–0.95; *P* = .033).

**Table 3:**
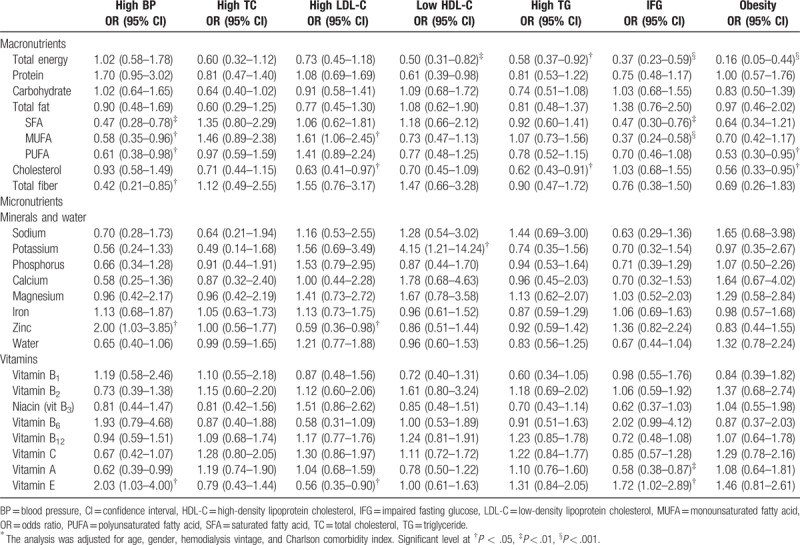
Odds ratios of having traditional cardiovascular risk factors among hemodialysis patients consumed adequately macronutrients and micronutrients^∗^.

Table 4 summarizes the associations between adequate dietary intake and nontraditional cardiovascular risks which show the nutrients relate to lower odds of low serum calcium are iron intake (OR, 0.42; 95% CI, 0.20–0.92; *P* = .031); to lower odds of hyperparathyroidism are MUFA intake (OR, 0.62; 95% CI, 0.41–0.93; *P* = .020); to lower odds of hyperhomocysteinemia are cholesterol intake (OR, 0.53; 95% CI, 0.29–0.97; *P* = .040), sodium intake (OR, 0.36; 95% CI, 0.14–0.91; *P* = .030); to lower odds of high hs-CRP are total energy intake (OR, 0.59; 95% CI, 0.37–0.93; *P* = .024), phosphorus intake (OR, 0.33; 95% CI, 0.17–0.63; *P* = .001); to lower odds of hypo-albumin are MUFA intake (OR, 0.27; 95% CI, 0.14–0.51; *P* < .001), PUFA intake (OR, 0.37; 95% CI, 0.18–0.77; *P* = .007), and water intake (OR, 0.26; 95% CI, 0.11–0.63; *P* = .003); to lower odds of inadequate nPNA are PUFA intake (OR, 0.27; 95%CI, 0.11–0.65; *P* = .003).

**Table 4:**
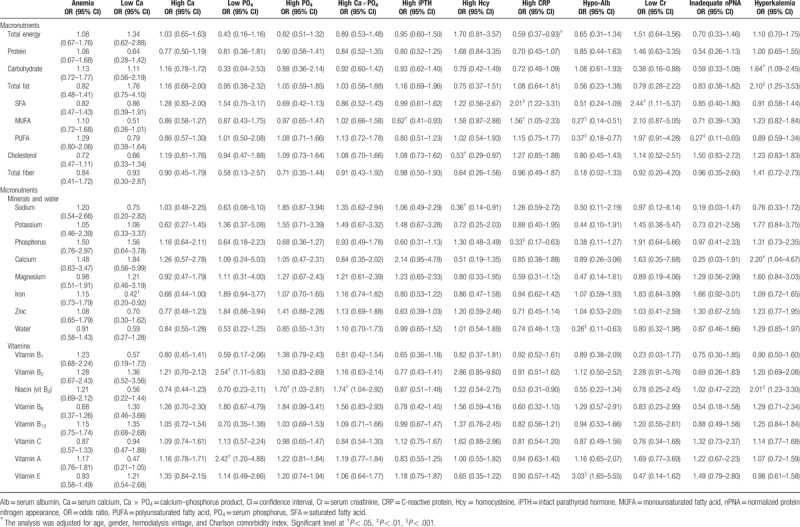
Odds ratios of having nontraditional cardiovascular risks among hemodialysis patients consumed adequately macronutrients and micronutrients^∗^.

## 4 Discussion

The present study finds the low prevalence of adequate intake of energy, protein, carbohydrate, fat, fiber, minerals, and vitamins, which contribute more evidence to previous findings that the hemodialysis patients have poor intake and do not meet the recommendation for renal-specific dietary intake.^[31,32]^

The percentages of patients with adequate intake of protein (27.8% vs 31.4%) and carbohydrate (54.9% vs 94.3%) in present study are lower than in the previous study, but the adequate intake of fat (15.2%) and fiber (8.7%) in current study is higher than previous one in the United States with 7.1% and 2.9%, respectively.^[11]^ In Turkey, 98.9% and 100% patients consume dietary total fat, and saturated fatty acids higher than recommendation level.^[33]^

The proportion of patients with high TC (18.7%), high LDL-C (48.4%), and low HDL-C (65.9%) are higher than those in the previous study with the proportion of high TC of 7.5%, high LDL-C of 4.3%, low HDL-C of 41.9%, respectively. The percentage of patient's high TG (42.7%) is similar to the previous one with high TG of 43.0%.^[33]^

Total energy intake shows the protective relationship in lowering the prevalence of low HDL-C, high TG, diabetes, obesity, and high hs-CRP. The findings are consistent with literature that adequate dietary energy intake, protein, and carbohydrate can reduce the risk of cardiovascular risk factors in hemodialysis patients.^[5,24,27,29]^ The present study, however, does not show the significant association between protein, carbohydrate intake, and cardiovascular risk factors. More studies in this area are required to carefully examine the association and effect of protein, and carbohydrate intake on CVD risk factors.

There was no significant difference found between fatty acid intake and TC, LDL-C, HDL-C, and Hb as the CVD risk factors in hemodialysis patients,^[34]^ which are also observed in the present study. However, adequate intake of SFA, MUFA, and PUFA significantly associated with a lower percentage of hypertension, diabetes, obesity, and hyperparathyroidism, hypoalbumin, and inadequate nPNA. The total fiber is significantly associated with the lower proportion of hypertension, this is supported the previous finding that substituting unsaturated fatty acid or fiber for saturated fatty acid can improve the serum cholesterol parameter.^[11]^

Regarding the nontraditional risks, the prevalence of malnutrition is low in the present study population with 11.4% hypoalbumin, it is much lower than the previous study with 44.1% hypoalbumin.^[33]^ This could be explained by the high quality of dialysis in Taiwan, and its successful application of universal health coverage in dialysis care which the expenditures were totally covered by national insurance. In addition, the mineral metabolic abnormalities are relatively high, the prevalence of anemia and inflammation are very high, and the extremely high prevalence of hyperhomocysteinemia.

In the present study, adequate dietary phosphorus intake is significantly associated with lower prevalence inflammation marker (hs-CRP), but not with serum phosphorus, or calcium–phosphorus product. In literature, a previous study elucidated a link between dietary phosphorus and serum phosphorus concentration and CVD event in dialysis patients.^[35]^ Sodium is associated with lower prevalence of hyperhomocysteinemia in the present study, which contributes to evidence regarding adequate sodium intake in managing hemodialysis patients.^[27]^ Adequate intake of zinc shows the significant association with a lower proportion of high LDL-C in the present study, which is consistent with the previous study that zinc is negatively associated with LDL-C in HD patients.^[36]^

In hemodialysis patients, vitamin B was shown to improve the cardiovascular outcomes.^[37,38]^ A randomized trial concluded that folic acid and vitamin B complex significantly reduced homocysteine and hs-CRP levels, and increased the serum albumin.^[39]^ However, in other studies, vitamin B shows no effect on the risk of CVD or death.^[40,41]^ In the present study, vitamin B significantly associates with low serum phosphorus, high serum phosphorus, high calcium–phosphorus product, and hyperkalemia, but vitamin B group does not show the association with other CVD risk factors, the result contributes to the evidence for clinical practice.^[42]^ This suggests more attention on the specific subgroups or different level of CVD risk factors to have more precise decision on the vitamin B therapy. Especially in the situation of a high proportion of patients with insufficient dietary vitamin B intake in the current and the previous study.^[33]^

Regards to fat-soluble vitamins, the previous study showed that the prevalence of sufficient vitamin D intake was 3.4%,^[43]^ slightly higher in the present study with only 3.1%. Multiple small trials have demonstrated the inconsistent benefits of vitamin D in dialysis outcomes (inflammation and anemia).^[44]^ In addition, the previous study demonstrated the positive effect of antioxidants (vitamin E) in reducing cardiovascular risks.^[37]^ In the present study, vitamin A is found as a protective factor for diabetes, the significant impact of vitamin E was observed on LDL-C. There is still lack of study regarding the role of micronutrients (especially fat-soluble vitamins) on CVD risks in hemodialysis patients. While the prevalence of adequate intake is reported very low in the present study, it raises an alarm in clinical practice in nutritional interventions in dialysis care in Taiwan and other countries. Despite the absence of clinical trials showing the benefit of vitamins intake on hemodialysis outcomes, encouraging patients to follow the dietary guidelines is important.^[10,33]^

There is a number of strengths and limitations in the present study that the interpretation of results should be cautious. Firstly, the study population is of good nutritional status, only 11.4% hypoalbumin, while albumin has been seen as the prominent biomarker of overall nutrition status, and correlated with other markers.^[25]^ Therefore, the associations between many dietary nutrients intake and CVD risk factors are not well explored in the present study. The second limitation is the nature of a cross-sectional study that limits the causality. However, in the absence of evidence from randomized controlled trials, the results analyzed by epidemiologic tools and methods, and reliable laboratory data could contribute to literature, and raise the awareness of nutritional regime in hemodialysis patients. Thirdly, the interactions between nutrients are not examined as relative small sample size, for example, calcium and magnesium can interact with fatty acids to form insoluble soaps in the intestine, that can prevent the absorption of the dietary fat, especially saturated fat, as a risk of high serum cholesterol and other CVD risks.^[45]^ Finally, the associations must be evaluated in the future prospective studies, randomized trials, and in different subgroups.

## 5 Conclusion

The present study provides the comprehensive view on macronutrients, micronutrients, and traditional, nontraditional CVD risk factors among hemodialysis patients. The percentage of patients with the adequate intake is significantly low, while the prevalence of CVD risks is remarkably high in hemodialysis patients. The study highlighted that adequate dietary nutrient intake associated with up to 84% lower risks of development of CVD, in turn, prevents the CVD diseases and death. This suggests that nephrologists, nurses, and dietitians need to educate patients to follow the dietary intake guidelines. The longitudinal design and randomized control trials are required in the future studies.

## Acknowledgments

The authors express the appreciation to medical staff and patients from Taipei Medical University Hospital, Wan-Fang Hospital, Shuang Ho Hospital, Cathay General Hospital, Taipei Tzu-Chi Hospital, Wei-Gong Memorial Hospital, and Lutong Poh-Ai Hospital. The authors also thank Chi-Sin Huang, I-Hsin Tseng, Yi-Wei Feng, and Tai-Yue Chang for helping with data collection.

## Author contributions

TVD consulted a statistician, analyzed the data and drafted the manuscript. TCW contributed to research methods and discussion. CTS, HHC, TWC, THC, YHH, SJP, KLK, HCL, and ETL contributed to study design and data collection. SHY contributed to overall study design and reviewed the manuscript. All authors read and approved the final version of the manuscript.

**Conceptualization:** Tuyen Van Duong, Te-Chih Wong, Tzen-Wen Chen, Tso-Hsiao Chen, Yung-Ho Hsu, Sheng-Jeng Peng, Ko-Lin Kuo, Hsiang-Chung Liu, En-Tsu Lin, Shwu-Huey Yang.

**Data curation:** Tuyen Van Duong, Te-Chih Wong, Chien-Tien Su, Hsi-Hsien Chen, Tso-Hsiao Chen, Yung-Ho Hsu, Sheng-Jeng Peng, Ko-Lin Kuo, Hsiang-Chung Liu.

**Formal analysis:** Tuyen Van Duong.

**Investigation:** Ko-Lin Kuo, Hsiang-Chung Liu, En-Tsu Lin.

**Methodology:** Tuyen Van Duong, Te-Chih Wong, Chien-Tien Su, Hsi-Hsien Chen, Tzen-Wen Chen, Tso-Hsiao Chen, Yung-Ho Hsu, Sheng-Jeng Peng, Ko-Lin Kuo, Hsiang-Chung Liu, En-Tsu Lin, Shwu-Huey Yang.

**Project administration:** Shwu-Huey Yang.

**Resources:** Shwu-Huey Yang.

**Software:** Tuyen Van Duong.

**Supervision:** Shwu-Huey Yang.

**Writing – original draft:** Tuyen Van Duong.

**Writing – review & editing:** Tuyen Van Duong, Shwu-Huey Yang.
